# Editorial: Risk factors in multiple myeloma identified before and during treatment: are we ready to personalize treatment?

**DOI:** 10.3389/fonc.2023.1247808

**Published:** 2023-07-31

**Authors:** Mattia D’Agostino, Carolina Terragna, Mark van Duin

**Affiliations:** ^1^ SSD Clinical Trial in Oncoematologia e Mieloma Multiplo, Division of Hematology, University of Torino, Azienda Ospedaliero-Universitaria Città della Salute e della Scienza di Torino, Torino, Italy; ^2^ IRCCS Azienda Ospedaliero-Universitaria di Bologna, Istituto di Ematologia “Seràgnoli”, Bologna, Italy; ^3^ Department of Haematology, Erasmus Medical Center, Rotterdam, Netherlands

**Keywords:** multiple myeloma, prognosis, risk assessment, minimal residual disease, liquid biopsy

Thanks to a better knowledge of Multiple Myeloma (MM) biology, new prognostic tools are available. Moreover, we are now able to induce unprecedented rates of deep remissions, which can be dissected with very high sensitive techniques, developed to detect very low levels of minimal residual disease (MRD).

However, treatment guidelines do not recommend risk-adapted approaches, stating that more evidence is needed to perform personalized medicine strategies. Thus, first-line therapy is still largely tailored on the eligibility of the patients to undergo autologous stem cell transplantation (ASCT) rather on either the biological characteristics of the disease itself or the deepness of response achieved.

In this Research Topic we tried to collect articles generating/reviewing data on factors detected both before and throughout treatment, that can enable a more precise prediction of MM patients’ outcome that would allow personalized approaches.

The first challenge has been considered the definition of high-risk disease, intended as disease with higher risk of relapse and/or death. Marcon et al. tried to reach a consensus on the definition of high-risk MM. A questionnaire was submitted to 6 Italian experts from the European Myeloma Network (EMN) and a consensus was reached, according to the Delphi method. Two rounds of the survey identified that the current risk stratification algorithm at diagnosis (R-ISS, Revised International Staging System) can be improved by adding chromosome 1 abnormalities, TP53 mutation, circulating plasma cells and extramedullary plasmacytomas.

Indeed, another work performed by You et al. supported the role of 1q21 gain/amplification as a high-risk marker in newly diagnosed MM patients. In this work, a multivariate analysis performed in 248 patients treated in a single center in China, showed that 1q21 was an independent adverse predictor of progression-free survival and overall survival.

Beyond first-line treatment, prognosis prediction in relapsed and/or refractory MM (RRMM) patients could be influenced by different factors, as compared to NDMM patients. Morabito et al. analyzed 919 RRMM patients receiving Elotuzumab-lenalidomide-dexamethasone or Carfilzomib-lenalidomide-dexamethasone. Interestingly, factors that can only be measured in RRMM like the interval from diagnosis to therapy, the number of previous lines and prior lenalidomide exposure were independently associated with the risk of death.

Another type of prognostic factor is MRD, which describes the disease dynamics during treatment. MRD measurement in the bone marrow can provide insights into disease status and might represent a fast method to evaluate efficacy in clinical trials. Flow cytometry is a solid MRD detection method, which is overall considered easy of use and returns quick results. A standardization of both flow cytometry panels and number of cells to be measured resulted in the development of next generation flow cytometry (NGF) by the Euroflow consortium. In addition to NGF, next generation sequencing (NGS) is considered a reference method for MRD detection. Both NGF and NGS can achieve sensitivity of <10^-5^ (1). The method commonly employed in the context of clinical trials for NGS-MRD detection has been the Adaptive ClonoSEQ™ assay. Despite the usefulness of this assay and its widespread use, alternative NGS methods might be considered, particularly when quick results are needed and budget is limited. In this Research Topic, Ferla et al. described a LymphoTrack-MiSeq platform for NGS-MRD. MRD was evaluated in 24 patients at day +100 post-ASCT. Among 11 patients reaching stringent complete remission, 5 were MRD negative and 6 positive by NGS performed according to the LymphoTrack assay. Despite the small sample size, MRD relation with prognosis was confirmed, as well as the feasibility of the method, including the use of a specific analysis software. MRD status can also be used to adapt treatment. Indeed, one report has indicated that patients with a positive MRD test during maintenance might benefit from treatment intensification (2). Many MRD-adapted trials are evaluating this kind of response-tailored approach.

However, the measure of MRD in the bone marrow is subjected to some limitation such as the invasiveness of the procedure and the spatial heterogeneity of the disease.

Indeed, liquid biopsy might overcome the shortcoming of bone marrow (BM) sampling and has an added value over conventional “single-site” BM biopsies, by avoiding sampling biases due to the limited glance on the molecular profile of the disease. MRD assessment by liquid biopsy in MM is indeed an innovative tool for disease characterization and disease dynamics monitoring.

In this Research Topic, Ye et al. reported the results of a meta-analysis of data available so far on the clinical role of cell-free (cf) DNA in MM, derived from seven studies, including 235 patients. Main observations concern the relevance of cfDNA for Minimal Residual Disease (MRD) detection and the role of both cfDNA and cfDNA tumour fraction (ctDNA) as prognostic factors.

The analysis of IgH rearrangement in cfDNA proved highly specific but not sensitive enough, as compared to the same analysis on BM aspirate (3). Nevertheless, a reliable comparison between the two approaches should be performed when multiple molecular targets will be employed to measure residual disease by ctDNA, as this expedient would increase the probability to detect low amount of ctDNA, suggestive of the residual presence of disease in the BM.

To date, the main added value of cfDNA analysis probably relies on the prognostic significance of the presence of peripheral molecular markers. In particular, the amount of detected cfDNA at diagnosis has a prognostic relevance (3), even though it is worth to note that ctDNA, deriving directly from the BM tumour burden, is more significantly associated to inferior OS. In addition, qualitative genomic information obtained from ctDNA might be more informative on disease distribution than the conventional single-site BM aspirate. Therefore, by pooling data from comparable studies, the present meta-analysis highlights the role of cfDNA as a good alternative to, or an important implementation of BM biopsies for monitoring disease dynamics in MM.

In conclusion, the articles in this Research Topic showed how risk factors identified at baseline (both at diagnosis and at relapse) and during treatment (both in the bone marrow and in the peripheral blood) can identify myeloma patients with different outcomes, paving the way to the design of personalized therapeutic strategies.

## Author contributions

All authors listed have made a substantial, direct, and intellectual contribution to the work and approved it for publication.
